# Supplementary material for: The Dual Prey-Inactivation Strategy of Spiders—In-Depth Venomic Analysis of Cupiennius salei
Source: Toxins (Basel). 2019 Mar 19;11(3):167. doi: 10.3390/toxins11030167 (PMC6468893; doi:10.3390/toxins11030167)
Supplement: Supplementary file 1 [file toxins-11-00167-s001.zip › Supplementary Fig S1.1-S1.19/Supplementary Fig S1.14.pdf]

Supplementary Fig S1.14

CLUSTAL O(1.2.4) multiple sequence alignment: Cystatins

```
CST_1_CUPSA                     MVMQLLVY--VFTACLFQASVGQMVGGWTPVEDTNSSDFQKITDFAAEKITSELRSNYKM
CST_2_CUPSA                     MVMQLLVY--VFTACLFQASVGQMVGGWTPVEDTNSSDFQKITDFAAEKITSELRSNYKM
CST_ALOMA                       MLVKLLIF--FFTACLFQAVVCQMVGGWSDVQDTNSEDFQKILSFATNEISVRLGSSNKK
CST_VIRFA                       --MRLLMFLCIVITSVVLKSAAILVGGWSEVSDVNSTEFRGIADFAVSKISESSNSPLRF
tr_A0A087UQM5_STEMI             --MKSIVI--IFVAGLSVGLAAVMTGGWSRAETTNSPSTKKYAEFAVAKISEELDSLFHM
gi_XP_015913031.1_PARTE         MSMHLNIF---ILFCMLSISYSMMTGGWTPVSDDNP-DAHEHAKFAARKISASTNNLYHM
                                  ::  :    .   :       :.***: ..  *  . :   .**. :*:    .  :

CST_1_CUPSA                     RLMEILSAKKQLVAGMKYRFEMRLGATECKVKDN--ED-GSLCDFLHCGAVHTCNATVWT
CST_2_CUPSA                     RLMEILSAKKQLVAGMKYRFEMRLGATECKVKDN--ED-GSLCDFLHCGAVHTCNATVWT
CST_ALOMA                       QVVEVLSAKKQVVAGINYKIAMKIGETNCKVDEN--DL-AKDCNLKPNGETHKCTAVVWH
CST_VIRFA                       KFIDVQNAIKQIVAGLNYKFTVIMGTTECKKSDQ--HD-ISECPFLHCGSWEKDSMH---
tr_A0A087UQM5_STEMI             KLMEILSAQKQVVSGMKYMLRMRIGATECEKDNN--EVDLSSCEFRDCEAPMTCNATVLD
gi_XP_015913031.1_PARTE         KVTNVSNIRRQVVSGLNYKMTITLAPTECKKNQNVSRDEIENCPLLLCKAPLSCNVNLWV
                                :. :: .  :*:*:*::* : : :. *:*: .::      . * :       . .

CST_1_CUPSA                     RPWLSDTR--LTNFSCRKRKGS--C
CST_2_CUPSA                     RPWLSDTR--LTNFSCEKEKGS--C
CST_ALOMA                       RGWLNDTR--LTDFSCEKEENT---
CST_VIRFA                       ----------VAIESCKNEPKS--C
tr_A0A087UQM5_STEMI             QAWKEDG------------------
gi_XP_015913031.1_PARTE         QAWLKEDGTKLTKSTCTPLPVDSNC
```

Percent Identity Matrix - created by Clustal2.1

1:	CST_1_CUPSA	100.00	98.53	52.59	37.40	48.33	33.33
2:	CST_2_CUPSA	98.53	100.00	54.07	38.21	48.33	33.58
3:	CST_ALOMA	52.59	54.07	100.00	31.97	35.00	29.32
4:	CST_VIRFA	37.40	38.21	31.97	100.00	31.82	35.54
5:	tr_A0A087UQM5_STEMI	48.33	48.33	35.00	31.82	100.00	31.09
6:	gi_XP_015913031.1_PARTE	33.33	33.58	29.32	35.54	31.09	100.00

Proteomic coverage trypsin

>CST_1_CUPSA cystatin isoform 1

MVMQLLVYVF TACLFQASVG QMVGGWTPVE DTNSSDFQK**I TDFAAEKITS ELR**SNYK**MRL MEILSAKKQL**
VAGMKYRFEM RLGATECK**VK DNEDGSLCDF LHCGAVHTCN ATVWTRPWLS DTR**LTNFSCR KRKGSC

>CST_2_S1_CUPSA cystatin isoform 2

MVMQLLVYVF TACLFQASVG QMVGGWTPVE DTNSSDFQK**I TDFAAEKITS ELR**SNYK**MRL MEILSAKKQL VAGMKYR**FEM
RLGATECK**VK DNEDGSLCDF LHCGAVHTCN ATVWTRPWLS DTRLTNFSCE KEKGSC**

Proteomic coverage chymotrypsin

>CST_1_CUPSA cystatin isoform 1

MVMQLLVYVF TACLFQASVG QMVGGWTP**VE DTNSSDFQKI TDFAAEKITS ELRSNYKMRL MEILSAKKQL VAGMKY**RFEM
RLGATECKVK DNEDGSL**CDF LHCGAVH**TCN ATVWTRPW**LS DTRLTNF**SCR KRKGSC

>CST_2_S1_CUPSA cystatin isoform 2

MVMQLLVYVF TACLFQASVG QMVGGWTP**VE DTNSSDFQKI TDFAAEKITS ELRSNYKMRL MEILSAKKQL VAGMKY**RFEM
RLGATECKVK DNEDGSL**CDF LHCGAVH**TCN ATVWTRPW**LS DTRLTNF**SCE KEKGSC

Supplementary Fig S1.14 Multiple sequence alignment of CST from the venom glands of *C. salei* (CST_1_CUPSA [MH766637]; CST_2_S1_CUPSA [MH766638]; CST_2_S2_CUPSA [MH766639]), *A. marikovskyi* (CST_ALOMA), *V. fasciatus* (CST_VIRFA), *S. mimosarum* (tr_A0A087UQM5_STEMI), and *P. tepidariorum* (gi_XP_015913031.1_PARTE). Signal peptides are marked in black characters, cysteines in white with a black box, positively charged amino acids in pink, negatively charged amino acids in blue, hydrophobic amino acids in red and polar amino acids in green. A putative proteinase inhibition site is highlighted in yellow.
